# Trauma and psychosis: a qualitative study exploring the perspectives of people with psychosis on the influence of traumatic experiences on psychotic symptoms and quality of life

**DOI:** 10.1186/s12888-022-03808-3

**Published:** 2022-03-24

**Authors:** Carolina Campodonico, Filippo Varese, Katherine Berry

**Affiliations:** 1 School of Psychology and Computer Science, University of Central Lancashire, Preston, Lancashire UK; 2 Complex Trauma and Resilience Research Unit, Greater Manchester Mental Health NHS Foundation Trust, Manchester Academic Health Science Centre, Manchester, UK; 3 Division of Psychology and Mental Health, School of Health Sciences, Faculty of Biology, Medicine and Health, Manchester Academic Health Science Centre, The University of Manchester, Zochonis Building, room 2.43, M13 9PL, Manchester, UK

## Abstract

**Background:**

Despite experiencing high rates of trauma and trauma-related conditions, people with psychosis are seldomly asked about possible traumatic events. While there are some barriers to discussing trauma in clinical services, research has shown that disclosure is not only possible but also beneficial to both psychotic and traumatic symptoms. The current study is the first to evaluate service users’ perception of the influence of trauma on the development and maintenance of their psychotic symptoms, as well as their views on how their life and mental health have been affected by traumatic events and their disclosure (or lack of).

**Methods:**

Eleven participants with experiences of psychosis and trauma took part in semi-structured interviews.

**Results:**

Consistently with previous literature, our participants reported high rates of interpersonal trauma, but had rarely had the opportunity to discuss any of these events. Using thematic analysis, we identified three major themes that have important implications for healthcare: factors that facilitate or hinder talking about trauma; consequences of talking or not; and relationship between trauma and psychosis. Participants generally benefited from talking about trauma and concerningly often associated the prolonged lack of opportunities to discuss traumatic events with negative feelings towards the self and with a deterioration of their mental health. Participants also recognised direct links between past traumas and the content and characteristics of their psychotic experiences.

**Conclusions:**

Our findings highlight the importance, as perceived by service users, of discussing trauma and looking at psychosis through a “trauma lens”. These results stress the need to systematically assess trauma history and traumatic symptoms in psychosis and might potentially help to overcome clinicians’ worries about discussing trauma with service users. Our findings underscore the need to change current practice and implement trauma-informed approaches to understand clients’ difficulties and provide support.

**Supplementary Information:**

The online version contains supplementary material available at 10.1186/s12888-022-03808-3.

## Introduction

People with experiences of psychosis report high rates of trauma [1] and are often diagnosed with trauma-related conditions such as post-traumatic stress disorder (PTSD) [2]. Repeated childhood trauma, which is particularly prevalent in people with psychosis [3], has frequently been associated with the new diagnosis of complex PTSD (CPTSD) [4], although research on psychosis and CPTSD is still in its early stages. Research has shown that exposure to adverse life events not only can result in these trauma-related conditions, but it can increase vulnerability to psychotic disorders and psychotic-like experiences [5], with a dose-response relationship where a greater number of exposures results in stronger risk for psychotic outcomes [6]. Aiming to clarify the relationship between trauma and psychosis, researchers have proposed different mechanisms that might explain this association.

The “affective pathway to psychosis” [7] suggests that mood-related symptoms such as emotional dysregulation, anxiety and depressive symptoms, might be determining factors for paranoid thinking. According to this hypothesis, anticipation of threat might result from anxiety, while precursors of psychotic symptoms, such as negative schemas about the self and self-esteem, can be negatively affected by depressive symptom [8]. These mood-related symptoms not only precede the psychotic onset, but can also negatively impact functioning [9] and precipitate relapse [10] in people experiencing psychosis and with a hystory of trauma. Recent evidence supports the idea that anxiety and moods might mediate the association between adversities and psychosis [11], while meta-analytic data found that, in people with psychosis, neglect and abuse positively associate with higher severity of depressive symptoms, further stressing the role played by mood symptoms in those with trauma [12].

Offering a different perspective, Morrison et al. [13] suggest that psychotic and traumatic symptoms are caused and maintained by similar psychological mechanisms and fall on a continuum of trauma-related reactions. In support of this theory, research has shown that hallucinations can be considered a form of post-traumatic intrusion, where the content of psychotic symptoms relates to the traumatic experiences or to the feelings of humiliation, fear and guilt associated with them [14, 15]. Hallucinations and other positive symptoms of psychosis have also been linked to dissociation [16, 17], another trauma-related response, with researchers suggesting that several symptoms of psychosis are in fact a dissociative phenomenon [18, 19]. Another type of psychotic symptoms that have been associated with traumatic reactions are delusional beliefs, as paranoia and PTSD following trauma have been found to share similar cognitive underpinnings [20]. Some authors have recently suggested that some people with psychosis might also experience a particular traumatic reaction described as “psychosis-related PTSD” (PR-PTSD), where the symptoms of PTSD are caused by events related to the diagnosis and treatment of psychosis (e.g. sectioning) or by the psychotic symptoms themselves [21]. However, despite increasing evidence about the existence of PR-PTSD [22], this is yet to be accepted as an official diagnostic sub-type.

The evidence of a relationship between trauma and psychosis has led to the inclusion of recommendations to assess PTSD in national clinical guidelines for the management of psychosis [23, 24]. However, trauma-history and traumatic stress symptoms are often unrecognised [25, 26] and inconsistently treated [27], possibly due to a lack of trauma screening within routine services or to a minimisation of trauma by the individuals themselves [2]. A systematic review found that most people who use mental health services are never asked about traumatic experiences such as childhood abuse and neglect, and that people diagnosed with psychotic disorders are asked even less than other service users [28]. Practitioners’ reluctance to enquire about trauma has been attributed to workload pressures and poor client engagement [29], and to concerns about offending or distressing the clients [30, 31].

However, clinicians’ worries about investigating trauma are not the only barriers to trauma disclosure in psychosis. A descriptive study investigating victimization found that 11% of participants with experience of psychosis would not report any type of victimization to anyone, and that in 57% of the cases patients would not report any victimization even when the psychiatrists thought that their patients had been victimised [32]. These findings follow those of Jansen et al.’s [25], who conducted a qualitative study to examine service users' experience of childhood trauma in the early phase of psychosis. The results showed that many traumatic experiences that participants previously reported in questionnaires, were not discussed when interviewed about their life story. The authors suggest that participants did not recognise the traumas in their personal narratives because such events had been dissociated or were not seen as something that should be discussed with others. Not being able to recognise and discuss traumatic stress in people with psychosis is a cause of great concern, as traumatic life events and their consequences can lead to more severe clinical profiles, worse overall functioning, and lower remission rates when compared to patients who did not experience such events [33]. Further negative consequences include drugs misuse [34], suicidal ideation [35] and the creation of barriers to people’s engagement in mental health services that would otherwise facilitate recovery [36].

Despite clinicians’ concerns and individuals’ reluctance to disclose trauma, different treatments are available that specifically target traumatic symptoms in people who experience psychosis, such as trauma-focused cognitive behavioural therapy for psychosis (TF-CBTp) [37], eye movement desensitization and reprocessing (EMDR) [38] and trauma exposure therapy [39]. Recently published systematic reviews suggest that these interventions for PTSD in psychosis not only are safe [40, 41], but they effectively reduce negative beliefs associated with traumatic memories, intrusive images and thoughts, hypervigilance and avoidance [42]. Treating PTSD in psychosis not only addresses the traumatic symptoms, but can also improve self-esteem and decrease delusions, hallucinations, anxiety, and depression (van den Berg & van der Gaag, 2012). Consensus has been recently reached about 16 essential principles of trauma-informed care in psychosis, including training on trauma-informed care for all staff, the adoption of a person-centred approach and the creation of an empathetic and non-judgmental environment [43]. This consensus might facilitate the consistent delivery of these interventions [43].

In support of these findings, qualitative research shows that promoting discussion of traumatic events in people with psychosis, even when individuals are hesitant, is possible. A study conducted with participants with first-episode of psychosis (FEP) and PTSD found that, although people might initially experience difficulty acknowledging that a trauma has occurred, it is possible to enhance their willingness to talk about trauma by giving them enough time and control over how trauma memories are shared [44]. Another study investigating the perspectives of young people with PTSD and FEP found that 86% of the participants showed improvement in both their PTSD and psychotic symptoms after talking about trauma, and reported that all participants found disclosure to be beneficial and worthwhile [45]. These qualitative studies offered privileged access to service users’ experiences of trauma and disclosure, providing a unique depth of understanding which is difficult to gain from closed question surveys, and offering descriptive rather than predictive results [46]. These findings, although promising, need to be further supported by additional research looking at patients’ perspectives of trauma.

Considering the high prevalence of trauma research in FEP, investigating the experiences of service users who have been diagnosed for a longer time would offer a more complete understanding of patients’ perspective on the relationship between trauma and psychosis. Also, many qualitative studies in psychosis have so far focused on disclosure and recovery from psychotic symptoms, rather than specifically on traumatic symptoms or people’s beliefs around the role played by trauma in influencing the development and maintenance of psychosis. Although the debate about what kind of events can be classified as traumatic is still ongoing, a recent meta-analysis has found that events currently recognised as traumatic by diagnostic manuals (e.g., involving actual or threatened death, serious injury, or sexual violence) are associated with only slightly higher PTSD symptoms than non-traumatic stressors [47, 48]. In the present study, we explore service users’ experiences of trauma (without limitation to what participants could describe as traumatic), and we discuss their perspectives on how trauma and disclosure have affected participants’ life and current mental health condition.

## Methods

### Design

A qualitative approach was chosen to explore participants’ individual views, feelings and authentic experiences. Semi-structured interviews were used as they allow participants to freely disclose their thoughts without constraint and elaborate on their answers, and offer the researcher the opportunity to follow up on answers given by respondents in real-time, generating valuable conversation around a subject [46].

### Participants

A sample of eleven participants was purposively recruited to complete semi-structured interviews as part of a larger quantitative research on post-traumatic reactions in people with psychosis. Participants were recruited if: 1) aged 16 or above; (2) able to provide informed consent; (3) experiencing psychosis as confirmed by relevant mental health professional; (4) experienced at least a difficult life event as defined by the Trauma and Life Events Checklist [TALE; 49]. Participants were excluded if: 1) experiencing dementia and/or other organic disorders; (2) had insufficient English to understand and complete the assessment; (3) had an intellectual disability which would impact the ability to complete the assessment. To create a group as heterogeneous as possible, the subsample of participants for this study was systematically recruited to differ in terms of: 1) age; 2) gender; 3) ethnicity; 4) levels of trauma symptoms as measured by the International Trauma Questionnaire [ITQ; 50]; 5) type of trauma anchored to the ITQ.

#### Measures

The *Trauma And Life Events Checklist* [TALE; 49] is a 22-item self-report measure specifically designed for routine trauma screening in psychosis services. It includes a list of common traumatic or stressful life events, as well as an item where participants can discuss traumas not covered previously. For each event that is endorsed, participants are asked if it occurred more than once and at what age(s). Three additional items ask participants which events are still affecting them and to what extent, using a scale from “not at all (0)” to “extremely (10)”. Currently, the TALE is the only trauma checklist including psychosis-specific potentially traumatic events (e.g. traumatic reactions to psychotic symptoms, hospitalisations or unusual behaviours), showing moderate psychometric acceptability overall, with excellent convergent validity and reliability for sexual abuse [49].

The *International Trauma Questionnaire* [ITQ; 50] is a self-report assessment tool that evaluates whether someone meets the criteria for ICD-11 PTSD and CPTSD. It includes 12 items, 6 measuring PTSD symptoms (re-experiencing, avoidance, and sense of current threat) and 6 measuring DSO symptoms (negative self-concept, affective dysregulation, and disturbances in relationship). Each item is scored on a 5-point Likert scale ranging from “Not at all” (0) to “Extremely” (4), with possible totals for the PTSD and DSO symptoms subscales varying from 0 to 24. Impairment caused by PTSD and DSO symptoms is investigated through three items each. The criteria for PTSD are met when each relevant symptom scores at least 2 (moderately), and when functional impairment is also observed (at least one of the three items scores ≥2). The criteria for possible CPTSD are met when in addition to PTSD, the participant also presents at least moderate scores across each DSO symptom, as well as functional impairment. The ITQ has good diagnostic and psychometric properties and has been shown to effectively capture the distinction between PTSD and CPTSD [50].

### Procedure

This research was carried out in accordance with the Declaration of Helsinki. Participants were invited from a pool of individuals with lived experience of psychosis, recruited as part of a larger study that had received NHS Research Ethics Committee and Health Research Authority approval (reference number: 18/NW/0469). After completing the previous study, participants were contacted via phone and offered the possibility to take part in this research. The researcher explained the study and agreed with them on a mutually convenient time and location (e.g., charity or Trust premises, private room at the University of Manchester, the participant’s home) to take informed written consent and complete the interview. The semi-structured interviews lasted between 50 to 90 minutes and were conducted using a topic guide (Appendix A). The questions were developed in collaboration with the research team. The topic guide was piloted with service users to ensure it was worded in a way that was easy to understand and not too distressing, and that the content was meaningful and relatable for people with lived experience of psychosis. Due to the iterative process of data collection and analysis, it became apparent during the research process that it was necessary to explore ideas not originally on the topic guide, like participants’ opinion on what causes unusual experiences such as hearing voices. Interviews were audio-recorded using a digital recording device and then transcribed verbatim and anonymised at the earliest opportunity. Transcription was carried out by psychology students on placement who had been provided appropriate training and were then double-checked by the first author. Interviews were conducted until data adequacy was reached [51].

### Analysis

The data were input in NVivo 12. The six steps of thematic analysis were systematically followed during data management, coding, and theme development, to allow the recognition of patterns across data as well as the production of findings easily accessible to different audiences [46]. After transcription, the researcher familiarised herself with the data by reading through the interviews and making notes about items of interest. At this stage, similar content was grouped into codes. The first author conducted line-by-line coding using a mixed deductive and inductive analytical process, allowing for theoretical assumptions to be interpreted from previous research and to be grounded from the data [52]. CC coded several portions of interviews under the supervision of the other members of the research team, who have extensive qualitative and clinical experience. Three interviews were double coded by the other two authors (FV and KB) to make sure that the codes identified were valid, and new lines of enquiry were considered to combine the insight of the author handling the data (CC) and the authors with extensive methodological and clinical experiences (FV and KB). Emerging codes and categories, as well as the interpretation of key texts and potential new lines of enquiry, were discussed between the authors. This process allowed a combined insight of the researcher handling the data closely and members of the team with wider methodological and clinical perspectives. Provisional themes were created by grouping similar codes and by discussing them within the research team [53]. The overarching themes “factors that facilitate or hinder talking about trauma”, “consequences of talking or not” and “relationship between trauma and psychosis” were driven by assumptions and abstract concepts underpinning the data, according to a latent interpretative approach [52]. Codes and themes were repetitively revised as new data was collected and coded. Up to the point when no new codes were identified and data adequacy was reached, which in our study meant that all codes relating to the study questions had appeared at least once in two different transcripts, recruitment, data collection, and analysis occurred concurrently [46, 51].

### Reflexivity statement

The first author (CC) is a white, middle-class, non-disabled female PhD student with an interest and background in working with trauma and people experiencing psychosis. CC believes that psychotic symptoms are strongly influenced by life experiences, especially by those events which have a traumatic nature. CC does not consider all psychotic manifestations as something intrinsically negative that need to be eradicated, but rather as meaningful attempts, conscious or not, to deal with a previous trauma. The other members of the research team (KB and FV) are also white, middle-class academics conducting research looking at the impact of trauma on psychosis, which might have influenced CC’s understanding of the relationship between these two conditions. Although this study has been designed to investigate participants’ point of view on the relationship between trauma and psychosis, CC was aware that some participants’ might not think that such a relationship exists and she made sure to choose participants in a way that made the sample as heterogeneous as possible, rather than choosing participants depending on their perspective around trauma and their current mental health.

During both the data collection and analysis, CC attempted to acknowledge her preconceptions and personal feelings through constant discussion with the other team members. As mentioned above, all the participants in this study had previously taken part in previous research so CC had the opportunity to develop a positive working relationship with them. These relationships have given CC privileged access to people’s stories, as they may have felt more confident in mentioning traumatic events or personal opinions that they had never spoken about before. As a result of their past experiences, some participants had strong negative feelings towards professionals. The existing relationship between CC and the participants, as well as the fact that CC has no experience as a clinician, made it difficult in the beginning to look at all the stories with impartiality. This was also affected by the fact that CC knows that trauma should be routinely assessed in clinical practice, but it is not. However, the other two authors have extensive clinical experience and offered their points of view during the analysis phase.

A critical realist [54] approach was used to analyse the data, which combines ontological realism and epistemological relativity. In other terms, we appreciate that the world has a concrete reality besides human constructions of it, but we also recognise that our understanding of the world is necessarily limited by our perspectives and standpoints within it. We acknowledge that our interpretations of what participants discussed are influenced by concepts that are socially constructed (e.g., schizophrenia and PTSD), and that complete objectivity is impossible.

## Results

The demographics of the participants are displayed in Table 1. While Table 1. presents the traumatic event to which the ITQ was anchored, it is important to note that all participants reported complex and repeated trauma histories. On the TALE, participants reported having experienced an average of thirteen different traumatic events. Most of these events were repeated experiences of interpersonal abuse both in childhood and adulthood, including bullying, discrimination, aggression, or insults by a close person, or feeling unsafe and unloved during childhood. More than half of the participants had experienced childhood sexual abuse and two participants reported having experienced sexual abuse as adults. Almost all participants reported feeling scared or threatened by psychosis-related symptoms or by contact with mental health services. As observed in Table 1., most of the sample had ITQ scores suggestive of a PTSD or CPTSD diagnosis.

**Table 1:** Demographic and clinical characteristics of the sample

Name	Age	Gender	Ethnicity	Diagnosis	ITQ group	Traumatic event
Deborah	18-24	F	White	Schizophrenia	CPTSD	Childhood sexual abuse
Tom	25-29	M	Mixed	Schizophrenia	PTSD	Psychosis-related trauma
Walter	30-34	M	White	Schizophrenia	CPTSD	Childhood neglect
Daniel	45-49	M	White	Schizophrenia	no diagnosis	Multiple traumas
Luca	60-64	M	Mixed	Schizophrenia	CPTSD	Childhood neglect
Steven	40-44	M	White	Schizophrenia	CPTSD	Childhood neglect
Mary	40-44	F	White	Schizophrenia	DSO	Betrayal trauma
Vanessa	60-64	F	White	Schizophrenia	CPTSD	Bullied at work
Sylvia	40-44	F	White	Schizophrenia	CPTSD	Sexual abuse
Symon	40-44	M	Mixed	Affective psychosis	no diagnosis	Physical Assault
Aisha	55-59	F	White	Schizophrenia	DSO	Psychosis-related

Notes: The table and paper used pseudonyms rathern than actual participants' names; *ITQ* International Trauma Questionnaire, *CPTSD* Complex Post-Traumatic Stress Disorder, *PTSD* Post-Traumatic Stress Disorder, *DSO* Disturbances of Self-Organisation

After conducting thematic analysis, three major themes were identified: 1. Factors that facilitate or hinder talking about trauma; 2. Consequences of talking or not; and 3. Relationship between trauma and psychosis. The following themes describe participants’ experiences and their perspectives on what has facilitated or hindered trauma disclosure in the past and what could help to create the right environment in the future for discussing traumatic experiences. This is followed by a description of the most common consequences both in relation to talking and not talking about trauma, where disclosure normally resulted in positive outcomes and failure to discuss trauma led to isolation and the development of negative feelings towards the self. The last theme describes the relationship between trauma and psychosis as identified by the participants, in that participants recognised that not having the chance to share their traumatic stories aggravated their overall mental health and described how the content of their psychotic symptoms related to past traumas.

### Factors that facilitate or hinder talking about trauma

Of the many factors that played a role in participants’ willingness to talk about trauma, having people in their life that were trusted and considered willing to listen, played the most important role in terms of disclosure. Participants who had the opportunity to discuss trauma usually confided in family and support groups or tried to approach a professional*.* Disclosure to family and support groups was facilitated by perceiving a safe environment and being around someone trustworthy or who had been through similar events. Discussing trauma was also facilitated by having a supportive professional, who participants described as a person who is kind, patient and interested, who asks questions without being perceived as judgemental. Sylvia describes which characteristics in a professional facilitate trauma disclosure:

*“I think the good ones are kind. I think they take the time to understand what you are saying, and they don’t rush you and they just try and- personally I like when people ask me questions because I can go on- so I feel like when they ask you questions they are trying to understand, they are trying to make the effort. My new CPN is lovely, because she doesn’t make it feel like I am a burden.”*

Most participants revealed that on many occasions they wished somebody had asked them about their difficult life events, as this was often the only push they needed to be able to share their stories and feeling less alone. However, when they felt ready to talk about trauma, they often did not have the opportunity to do so, or other people seemed distracted and not interested. Disclosure to family was often met by negative reactions including anger, disbelief and dismissal, while disclosure to support groups was sometimes regarded as disappointing or insufficient. Daniel describes his mother violent reaction to his attempt at disclosing a sexual abuse:

*“I think I said to you, when I was trying to tell me mum about the sexual assault I said, I told her about the worst bit, and she came down and kicked the Jesus out of us.”*

During hospital admissions, participants felt that they had no opportunity to disclose because of a range of negative views of professionals (perceived as too busy or as “*the enemy*”). Similarly, two participants reported not having the opportunity to talk when they were on probation following a prison sentence, as people around them already had a negative opinion of them and were not interested in listening. Sylvia describes how she felt when she got sectioned and realised that staff in the hospital was not interested in investigating deeper causes for her behaviour:

*“it’s just weird cause you think you are getting into the hospital to be helped, but when you get there the only thing they are doing is just keeping you alive. They don’t talk to you about… they give you the medication and otherwise they sit in their office and do- whatever they do.”*

Among those patients who did not experience rejection, the fear that people would not believe them or would not understand the trauma or the participants’ ways of coping with it, was enough to prevent them from discussing the event. At other times participants were simply not ready to talk about trauma as they had not processed it themselves, either because they were unable to recall the traumatic events or because they had spent too much time denying what happened. Another common hindering factor consisted in being too scared of possible consequences of talking about trauma. Participants often believed that bad things would happen if they spoke about the traumatic event (e.g., threat to them or family members), either because the voices (i.e. auditory verbal hallucinations) or the perpetrator of the abuse told them so. Vanessa explains she never spoke about trauma as she feared negative repercussions on her parents:

*“He said if I ever told mum and dad, he’d make sure the police found out about their illegal poker game. They’d lose the house; they’d go to jail, and I’d end up in the orphanage. That is what he threatened. Well, when you’re nine years old you’d believe it.”*

### Consequences of talking or not

Participants identified many outcomes related to talking or not about trauma. Those who had the opportunity to discuss trauma reported experiencing positive consequences both for themselves and others. Many participants said that although talking about trauma is hard, when they were able to do so they felt as if a weight had been lifted off their chest. Similarly, some people remarked that discussing past experiences offers a chance of releasing negative emotions that would normally bottle up and become ‘negative energy’. Walter acknowledges the difficulties of discussing trauma, but appreciates the benefits that might come from it:

*“I think it’s helpful... like, it’s not nice reliving past pain, but when you talk through it, when you can talk through it and work your way through it maybe, it’s better than just remembering stuff and going through it every time.”*

Participants also found value in sharing their stories to help others feeling less lonely and desperate, and reported experiencing positive feelings when they thought they had been useful to someone else. In this respect, participants noted that knowing that someone else went through similar events, experienced similar feelings and survived, could be inspirational. For example, a participant said that the reason why they agreed to participate in this research, was that they hoped that their story would reach more people in similar situations. Luca explains his motivation for sharing his story:

*“But see… me, when I do this in here, in me own tinpot way, it’s my way of tryna give something back to somebody else’s. If from what I say, somebody else can make sense of it, and they go, ‘oh my god, that’s the way I think, that’s what I’ve heard’. It might just be one life or two lives or whatever, but it’s not just me. Because I’ve been through it, you know, like I’ve lost my daughter, I’ve lost my – you know – lost my best friend.”*

Some participants who had not yet had the chance to talk about trauma, said that they would welcome the possibility to do so as it could potentially help them changing perspective on what happened and fully understand the consequences trauma had on their lives. Participants reported that not having the opportunity to discuss trauma mostly impacted the way they made sense of it. Not having anyone to talk to influenced their understanding of the reasons behind their maltreatment, which meant that for a long time they felt like they were to blame. Self-blame and guilt added to the wide range of negative emotions that they were already experiencing in relationship to trauma. For example, not being able to discuss trauma also led to feelings of shame, as participants considered themselves weak for feeling scared, angry or lonely, especially if they thought that abuse was ‘normal’ or deserved and they had no reason to be feeling that way. It was proposed that this way of thinking about trauma and themselves might have eventually led to the inability to cope with the traumatic event. Vanessa describes how she felt when she was blaming herself for her mother’s death, and the relief she experienced when she could finally forgive herself:

*“It was anger, more than anything. I blamed myself when my mother died, I didn’t know her heart had burst. I just thought she died of a heart attack. I blamed myself for not calling the doctor, I blamed myself for not staying up all night later. But when I found out five years later that her heart had burst and there was nothing I could have done, I finally forgave myself. If they only had been honest, I wouldn’t have gone through all that grief.”*

Participants reported that not being able to discuss trauma had long-lasting consequences on their lives. They reported not feeling in control, because they often had to give up work and education, as trauma and its consequences affected their motivation and their ability to cope with daily tasks and be among other people. The idea of not having accomplished anything in life led to feelings of sadness and depression. Vanessa describes how the consequences of trauma made it impossible for her to hold on to a job:

*“I couldn’t work, because I’ll tell you why- I never know when I wake up what mood I’m going to be in, or when I’m going to wake up. Yesterday I had twelve hours sleep, the day before I had fifteen hours sleep! How can you go to work when you are like that? And if somebody looked at me the wrong way when I was upset, I’d burst into tears, and I couldn’t cope.”*

Interpersonal trauma was more frequently associated with avoidance of social contact, as participants considered their loneliness a result of their past life events. Not being able to discuss traumas resulted in being cautious around other people and keeping distance for fear of being hurt or losing someone they cared about, leading to increasing isolation. Participants said they suffered because of their social withdrawal, and even when they wanted to connect with others, they reported not knowing how to do it, as interpersonal traumas resulted in difficulty expressing and feeling emotions, and confusion around the meaning of love and affection. Mary explains that she realises that she is the one keeping people distant, but she does not know how to stop doing that:

*“And I can tell myself, I understand that I am- I’m doing it, I don’t let people inside in my heart. It’s because I feel like they’re gonna hurt me. I have got that and my head saying “They’re gonna hurt ya, they’re gonna hurt ya” or something. That is how I feel. Like I do not know how to not do that […] and I have no friends now, not a friend, and it’s pretty sad that – it makes me really lonely when I think about it.”*

### Relationship between Trauma and Psychosis

While almost all participants agreed that trauma impacted their whole life and that they were largely still affected by it, they had different thoughts around how it influenced their current mental health. While a few participants were unsure, many believed that trauma was the cause of their psychotic symptoms and psychosis-related diagnosis, and that if they had had the chance to discuss trauma earlier this could have prevented their current condition. One participant thought that professionals diagnosed them with psychosis only because they did not believe their trauma disclosure, and they were convinced that their life would have been much better if only they had received help when they were looking for it. Participants’ mental health slowly or suddenly deteriorated as a direct consequence of trauma, or as a result of ignoring the event and its effects for too long. Symon explains how having the chance to discuss trauma when it first happened, could have prevented their current mental health status:

*“I think the best time was in 1992 when it first happened. If I would’ve had someone to speak to then, perhaps I wouldn’t have the- I wouldn’t have the psychological damage.”*

Even when the trauma did not cause traumatic or psychotic symptoms, it shattered the participants’ confidence, coping abilities and mood, until they could not deal with daily tasks anymore and felt useless and hopeless. Feeling constantly scared as a consequence of trauma, as well as feeling continuously on the edge of a mental breakdown, wore participants down until something else traumatic happened and they could not cope anymore. Mary offers an example of how her everyday activities eventually became unmanageable:

*“Every tiny little thing that I did I- I’d phone my husband when he was at work like- I’d spill, do you know the tipp-ex, that white thing that you take the pen off… and I’d start panicking.”*

Participants recognised specific links between trauma and the content and characteristics of psychotic symptoms. For example, memories of the trauma faded into visual hallucinations, and voices that screamed and cried often sounded like the participants at the time the trauma happened. They sometimes recognised that feelings of suspiciousness and ‘paranoia’ were also linked to their trauma, and so were some voices warning them off every time they left the house. Deborah explains her understanding of the relationship between her past traumatic experiences and current mental health:

*“I think that’s why I hear voices, it’s because I was sexually abused […] and I can see how these different mental health experiences are really clearly linked to what happened to me, through like the content of my voices.”*

However, the voices were not always perceived negatively. Despite being daunting, some participants recognised that the voices were probably just trying to keep them safe and to avoid new traumas. The voices would get anxious when the participants tried to talk about trauma, or they would directly order the participants to not talk about the traumatic events. On the other hand, a participant reported appeasing the voices to be able to cope with trauma, and that once they started dealing with the traumatic memories the voices also got better. Walter described the protective role played by his voices:

*“I think they try to keep me safe. They’re not very nice, they tell me to hurt people or cars... how are you supposed to hurt a car I don’t know, but... psychosis could very well be linked to my troubled history... they’re just… the things I hear are trying to keep me safe from what... going through the pain again, I guess.”*

## Discussion

This study investigated service users’ perception of the role played by trauma in influencing the development and maintenance of their psychotic symptoms and their views on how traumatic experiences and their disclosure (or lack of) have affected their life and mental health. The study found that participants had high rates of interpersonal traumas and that discussion of these traumas could be facilitated by having appropriate conditions to do so. When provided with the right opportunity, mostly referring to having somebody trusted and interested in listening, talking about trauma usually led to positive outcomes for the participant and the people around them. On the other hand, not being able to discuss traumatic life experiences, together with feeling scared or not ready to disclose, affected the way participants made sense of the trauma and often led to negative feelings towards the self. Most participants believed that the prolonged lack of opportunities to talk about trauma aggravated their difficulties, and they recognised direct links between past traumas and the content and characteristics of their psychotic experiences.

Consistently with what found in the literature [2, 28], our participants reported seldomly being asked about trauma history or traumatic stress symptoms, despite national clinical guidelines recommend trauma assessment in psychosis [23, 24]. Our research provided a richer description of the personal implications of not having the occasion to talk about trauma, grounded in participants’ testimonies and personal experiences. In line with Tong et al.’s [44] qualitative research on FEP, our participants agreed that conversations about trauma can be uncomfortable, yet they welcomed the idea of a professional asking them about their experiences, as long as they felt safe and not judged. This supports the evidence that rates of disclosure are not influenced by patient characteristics [32], but rather by external factors such as the reactions of those to whom the traumas are disclosed. Our results not only further stress the importance of routine assessment of trauma in psychosis, but also match meta-analytical evidence that trauma disclosure is ultimately beneficial [55]. Negative reactions are particularly detrimental as not only they influence willingness to disclose, but they can also increase PTSD symptoms [56]. In our sample participants agreed that not talking was largely influenced by not having people in their life that were trusted and considered willing to listen, by previous negative reactions from family members or by poor relationships with professionals. Overall, our findings reflect and support the conclusions from qualitative research on trauma and FEP, suggesting that discussing trauma in psychosis is possible and beneficial.

Our findings that discussing trauma often resulted in positive consequences for the participants and others are in line with previous research suggesting that trauma disclosure can improve overall well-being [57]. For example, participants felt that telling their stories allowed them to process some of the associated memories and being able to use their experiences to help others get through similar events increased their self-esteem. These findings have important clinical implications as they suggest the value of using client narratives within services. By normalising and sharing positive experiences of patients who decided to talk about trauma, professionals can encourage disclosure. As both clinicians and service users gain confidence in the safety and benefits of discussing trauma, routine trauma inquiry initiatives would be facilitated.

The fact that participants who did not talk about trauma often blamed themselves for what was happening in their lives and felt weak for being scared or lonely, also finds support in the literature, which suggests that not being able to discuss trauma in psychosis can result in negative outcomes [33]. The role of self-blame here is particularly important, as research has found that in people with a history of trauma, internalising feelings of blame contributes to psychological distress [58] and has a deleterious impact on physical health [59]. Studies around self-blame in psychosis have so far focused mostly on caregivers rather than on service users [60]. Further research is needed to investigate the role of self-blame in relation to trauma and psychosis, to understand if self-blame arises as a result of prolonged non-disclosure or if the relationship is possibly more complex (e.g., bi-directional).

Similarly to previous research, participants reported that the content of psychotic symptoms was often related to their traumatic events and the negative feelings associated to them [14, 15]. When interviewed, participants reported experiencing many psychosis-related traumas, including being scared because unable to distinguish reality from fantasy, by their hallucinations or by going through several negative hospital experiences. These findings stress the need to move away from what is currently considered traumatic within the diagnostic classifications systems and adopt a wider and more ideographic understanding of what constitutes a traumatic experience. Events currently recognised as traumatic by diagnostic manuals (e.g., involving actual or threatened death, serious injury, or sexual violence) are associated with only slightly higher PTSD symptoms than non-traumatic stressors [47, 48]. Widening the definition of trauma could affect who is treated for trauma-related disorders, how treatment is understood and could reduce the risk of invalidating people’s attempts of disclosing trauma. If more people who experience psychosis were treated for trauma, it might impact how their symptoms are conceptualised, as we know that flashbacks of an event can be difficult to distinguish from hallucinations [14, 15] and that the way these intrusive experiences are labelled determines the diagnostic interpretation of these symptoms as either a function of psychosis or PTSD [61].

While there is extensive quantitative evidence on the potential links between trauma and psychosis, including re-victimisation [62], our findings indicate that the views of people with psychosis are sometimes, although not always, congruent with these research findings. Consistently with studies that found that re-victimization increases the likelihood of having psychotic experiences [63], our results suggest that feeling constantly scared as a consequence of trauma, or feeling continuously on the edge of a mental breakdown, led participants to a point where they were so worn down that they could not cope with anything anymore.

Our findings on the relationship between trauma and psychosis, and the fact the participants reported wanting to discuss trauma rather than just be treated for the psychotic symptoms, fit well within the recovery movement that advocates for a broader and more individualised understanding of what recovery in mental health means [64], as well as with increased calls for the implementation of trauma-informed approaches within mental health care [65]. In the context of trauma in psychosis, this would not only mean reducing the psychotic symptoms, but also trying to understand these symptoms as potential reactions to traumatic life experiences, and therefore benefit from general support consistent with trauma-informed care as well as, in some cases, trauma-focused therapy. Trauma-informed care in psychosis, based on knowledge and understanding of how trauma affects people's lives, have widely agreed on principles [43]. Trauma-focused therapy has shown promising results [66], with no evidence that it could lead to re-victimization or the exacerbation of PTSD or psychotic symptoms [67]. For our participants, not being able to discuss trauma was potentially related to the development of their psychotic experiences, which has been found to be the case in other mental health conditions, as non-disclosure has been associated with higher PTSD symptoms and depression [68]. Trauma-informed approaches and trauma-focused interventions could be particularly useful and more acceptable for these participants who already see a connection between their past traumatic experiences and their symptoms of psychosis. TF-CBTp in particular, has been found to have promising effectiveness, as supported by case-series [37] and feasibility trials [69]. Prolonged exposure and EMDR have also been found to be effective in reducing both symptoms of PTSD and psychotic symptoms in people with experiences of psychosis [70]. These types of interventions have shown to improve both service users' experiences and working environments for staff, as they foster understanding, respect and trust between patients and professionals and avoid the risk of service users being retraumatised by ‘trauma-uninformed’ staff [65].

### Limitations

It is important to remember that participants in this study had already contributed to previous research about trauma and were, therefore, more likely to be interested in the topic and willing to discuss their experiences, even if for the first time. This could potentially mean that those individuals who would not even consider discussing a traumatic event may not be represented and that additional challenges to disclosure could exist. It might also mean that the series of positive consequences associated with discussing trauma reported by our participants do not necessarily apply to all service users. Additionally, due to the diverse sample, potential moderating characteristics were not adequately represented to allow the exploration of subgroup differences (e.g., ethnicity and gender). As evidence indicates that minority ethnicities have a higher chance to experience coercive and potentially traumatic pathways into care [71], future qualitative research is needed to further our understanding of the experiences of trauma in minorities. Finally, while our results contribute to existing literature suggesting that many people with traumatic experiences and psychosis attribute their psychotic experiences to their traumas, we recognise that this study was not designed to test this putative mechanism nor any causal relationships. Although trauma seems to be one pathway to psychosis, there are people with experiences of psychosis who do not report any trauma [72].

## Conclusions

The findings from this research highlight the importance, as perceived by service users, of discussing trauma. Our results suggest that similarly to findings in non-clinical populations, people with psychosis are willing to discuss trauma and think that disclosure might be associated with positive outcomes. On the other hand, not being able to discuss trauma was normally associated with negative outcomes. Despite not having the opportunity to discuss trauma previously, service users welcome the idea of discussing trauma and even hope that doing so might improve psychotic symptoms. Future research is needed to systematically investigate the connection between trauma and psychotic symptoms and explore the benefits of discussing trauma also with service users who have been diagnosed for a long time. By looking at psychosis through a “trauma lens” and implementing trauma-informed approaches to understand clients’ difficulties and provide support, we might be able to see faster recovery and overall improved functioning and wellbeing.

## Supplementary Information

**Additional file 1.** Appendix A: Topic guide.

## Data Availability

The dataset generated and analyzed during this study are not publicly available as interview transcripts contain sensitive and potentially identifying information but are available from the corresponding author on reasonable request.

## Abbreviations

CPN: Community psychiatric nurse
