# Supplementary material for: Trauma and psychosis: a qualitative study exploring the perspectives of people with psychosis on the influence of traumatic experiences on psychotic symptoms and quality of life
Source: BMC Psychiatry. 2022 Mar 24;22:213. doi: 10.1186/s12888-022-03808-3 (PMC8944047; doi:10.1186/s12888-022-03808-3)
Supplement: Supplementary file 1 — Additional file 1. Appendix A: Topic guide. [file 12888_2022_3808_MOESM1_ESM.docx]

Appendix A: Topic guide

**How people with experience of psychosis view trauma and their responses to it.**

It is very common for people with experiences of psychosis to have lived some difficult life events in the course of their life, things like bullying, physical or sexual abuse, neglect.. **Although** anything could be considered traumatic or stressing and it varies person by person.

Last time we talked about some very personal experiences, and I was wondering if it would be ok today to discuss some of those more in depth. Is that ok with you? This could be quite stressing, so please feel free to ask for a break or interrupt the interview at any time.

Today you will be doing more of the talking, which is perfectly normal as what I am hoping for is to have a chat to grasp your opinion on few topics. We will talk about how trauma has affected your life (if at all), about how/if you coped with stress and if you think there are actually any positive consequences that would not be there if the difficult event did not happen.

**Warm-up questions:**

1. How have you been doing since last time?

**Trauma:**

**Last time we talked, you mentioned that some difficult events had an impact on your life.**

1. Which event/s (happened at any point in your life) do you think had an impact on you?

*PROBES: How was your childhood?*

*Refer to items on TALE or G12 on PANSS*

*Any chance you remember the ITQ event? Would you say that was worse than the events you just mentioned?*

1. Do you mind if I ask you something more about this? We do not have to discuss it if you do not want to.
2. How was your life affected by what happened?

***PROBES****: your capacity to work/ go to school? Your relationships or social life? Your physical health?*

**If not mentioned previously**: And how was your mental health affected? How does it link with the experiences you are having?

*PROBES: voices, believes..*

1. People have very different opinions on what causes unusual experiences such as hearing voices. Some people says it’s biology, some say drugs, other people say it’s stress or trauma. What is your opinion?

*PROBES*: Do you think stress could actually cause a person to experience mental health difficulties?

What was in your case?

1. How is the *event* or its consequences still affecting you now?

*PROBES: flashbacks and/or intrusive memories,* ***related psychotic symptoms***

1. Have you had the chance to talk about the *event* or any symptoms connected to it, with a mental health professional?

*PROBES: did your health professional ever asked you about trauma?*

**If yes**: How did you find it? Was it helpful?

**If not**: How do you think being able to talk about the trauma would impact your mental health? Do you think it would help? How?

1. Have you had the chance to talk about it with anybody else?

*PROBES:* *like a support group or people who had the same experiences?*

1. What do you think it distinguishes a good mh professional?

**Resilience:**

**There is no right or wrong way to deal with the effect of a trauma, and previous participants mentioned the importance of having family and friends around, some others just shut everybody out and that worked from them.**

1. How did you manage the emotional consequences of what happened?

*PROBES: e.g. social support, own coping abilities, being hopeful?*

1. What do you think was missing, that would have helped you to cope better, or that would have made the process easier/quicker?

*If they only mention negative coping, mention sth good they are doing (e.g. pick up a niece) and ask them what makes them able to do that*

1. Do you think people should learn earlier about mh?

**PTG:**

**Paradoxically, many people report that sometimes as a result of the trauma they end up experiencing some positive changes. It varies on person to person, and also depends on the type of trauma. Some people become closer to God, or they realised for the first time how strong they were..**

1. Do you think there was any change in your life, that you now value, and would not be there if the difficult event did not happen?

*PROBE:* *prompt them with their answers from the PTGI*

1. Do you think what happened help you to grow up as a person?

**If not**: Why do you think that was the case?

1. What do you think might make people able to experience positive changes after a trauma?

*PROBES: e.g. social support, own coping abilities, optimism?*

1. How does that make you feel in relations to what happened in the past, the fact that you might have gained something from the trauma?
2. Does it offer you a new perspective on what happened?

**Additional information:**

1. Would you like to add anything else? Is there anything that you think would be relevant that I have not asked about?

The interview questions are constantly evolving, so if you think there are e.g. better questions that I could ask people feel free to say so.

**Experience of interview:**

Can I ask you how you have found the interview today?

**Thank and pay participant!**
